# A Multi-Source Data Fusion Framework for Revealing the Regulatory Mechanism of Breast Cancer Immune Evasion

**DOI:** 10.3389/fgene.2020.595324

**Published:** 2020-11-12

**Authors:** Xia Chen, Yexiong Lin, Qiang Qu, Bin Ning, Haowen Chen, Lijun Cai

**Affiliations:** ^1^College of Computer Science and Electronic Engineering, Hunan University, Changsha, China; ^2^School of Basic Education, Changsha Aeronautical Vocational and Technical College, Changsha, China

**Keywords:** data mining, data fusion, correlation analysis, complex diseases, immune evasion

## Abstract

For precision medicine, there is an enormous need to understand the immune evasion mechanism of tumor development, especially when tumor heterogeneity significantly affects the effect of immunotherapy. Recognizing the subtypes of breast cancer based on the immune-related genes helps to understand the immune escape pathways dominated by different subtypes, so as to implement effective treatment measures for different subtypes. For that, we used non-negative matrix factorization and consistent clustering algorithm on The Cancer Genome Atlas RNA-seq breast cancer data and recognized 4 subtypes according to the curated immune-related genes. Then, we conducted differential expression analysis between each subtype of breast cancer and normal tissue of RNA-seq data from non-cancer individuals collected by the Genotype-Tissue Expression to find out subtype-related immune genes. After that, we carried out correlation analysis between copy number variants (CNV) and mRNA of immune genes and investigated the regulatory mechanism of the immune genes, which cannot be explained by CNV based on ATAC-seq data. The experimental results reveal that *CDH1* and *PVRL2* are potential for immune evasion in all 4 subgroups. The expression variations of *CDH1* can be mainly explained by its CNV, while the expression variation of *PVRL2* is more likely regulated by transcript factors.

## Introduction

Precision medicine is an emerging strategy for cancer prevention and treatment that takes into account individual variability of genetic basis for each patient (Ashley, 2016). With the help of next-generation high-throughput sequencing technology, researchers have become more and more familiar with the details of whole genome mutations, and the overall relationship between different omics data has become more and more systematic.

The Cancer Genome Atlas (TCGA) provides multi-omics data for global researchers to understand the onset and development of human cancers. For example, genomic and transcriptomic data of bulk tumor tissue samples play an important role in studying the tumor microenvironment (TME; Thorsson et al., 2018), and measures of immune infiltration define molecular subtypes of a number of different cancers. Although TCGA also collects non-malignant adjacent normal tissue samples of cancer patients, this kind of normal sample faces two main limitations: the matching normal sample size is too small, and the normal sample derived from cancer patients still cannot completely replace the real normal tissue sample from non-cancer individuals. Fortunately, the Genotype-Tissue Expression (GTEx) project collected 54 non-diseased tissue sites across nearly 1000 individuals including whole genome sequencing (WGS), whole exome sequencing (WES), and RNA-Seq data (GTEx Consortium, 2015). Therefore, studying the tissue-specific differential gene expression between TCGA cases and GTEx controls may help researchers to locate potential pathogenic genes (Mounir et al., 2019). ATAC-seq (Assay for Transposase-Accessible Chromatin using sequencing) can quantitatively measure the accessibility of genome-wide chromatin. Corces et al. (2018) generated the profile chromatin accessibility of 410 TCGA samples from 23 cancer types using ATAC-seq technique. This study revealed that the combination of ATAC-seq data and other omics data could help researchers find out the transcription factors and enhancers regulating pathogenic genes or immune genes.

Tumor heterogeneity means the molecular and cellular difference of a single tumor between different tumor patients (inter-tumor heterogeneity) or even different tumor formation sites in a single patient (intra-tumor heterogeneity) (Alizadeh et al., 2015; Jia et al., 2018). However, researchers only know the tip of the iceberg of tumor heterogeneity, resulting in a lack of targeted precision medical treatments. Breast cancer also shows heterogeneity both at molecular and cellular levels, which inhibits the effects of diagnostic, prognostic, or predictive strategies in routine clinical practice. Turashvili et al. conducted a comprehensive review on breast cancer both from inter-tumor heterogeneity and intra-tumor heterogeneity aspects (Turashvili and Brogi, 2017). Bou-Dargham et al. (2018) clustered breast cancer samples collected from TCGA with using 1356 immune-related genes as features, finding the dominate evasion mechanism in seven clusters. However, it is still unknown how a large part of the mechanism of tumor cells regulate the expression of immune genes to evade immune cell killing (Lowry and Zehring, 2017; Anmamed and Chen, 2018).

Aiming to understand the regulatory mechanism of breast cancer immune evasion, in this study we sought to identify differentially expressed immune-related genes in tumor tissue by comparing the TCGA and GTEx mRNA data. To find out the reasons for the variations of immune gene expression, we carried out correlation analysis between CNV and mRNA, and we also analyzed the relationship between transcript factor (TF) and target immune gene based on ATAC-seq data. Then, the relationships between TFs and immune gene were validated by common databases.

## Materials and Methods

### The Multi-Source Data Fusion Framework

**FIGURE 1:**
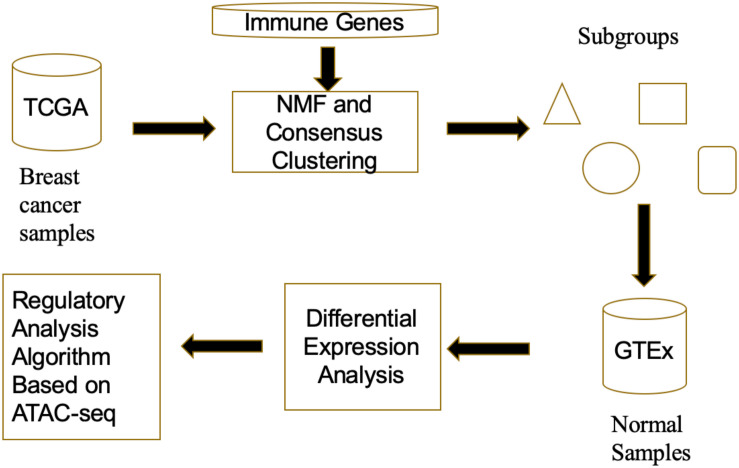
The flowchart of the multi-source data fusion framework.

In Figure 1, we depicted a proposed framework for investigating regulatory mechanisms of tumor immune evasion. The multi-source data fusion framework includes three main procedures: First, NMF clustering algorithm was used to recognize subgroups of TCGA breast cancer samples. Note that the immune-related genes are considered as clustering features, so that the subgroups may have different immune evasion pathways; Second, to avoid data bias of normal tissue collected from cancer patients, we compared the GTEx normal data with each subgroup of TCGA breast cancer samples, identifying differentially expressed immune-related genes. Finally, we designed a regulatory analysis algorithm to find regulatory factors for immune-related genes expression variations based on ATAC-seq data.

### Collecting Candidate Immune Genes

To understand how these tumor cells evade damage from immune cells (such as T cells, NK cells, and so on), we should first compare the differences of candidate immune-related genes in tumor tissues and normal tissues. Patel et al. (2017) conducted a comprehensive investigation on essential genes for cancer immunotherapy and provided gene-ranking based on sgRNA enrichment analysis. In addition, we also manually collected some important NK ligands. Therefore, we collected a total of 2171 candidate immune-related genes.

### Gene Expression Dataset Unifying TCGA and GTEx

Before comparing the gene expression difference between the tumor tissue from TCGA breast cancer and normal breast tissue from GTEx, several important issues should be addressed, such as uniform realignment, gene expression quantification, study-specific biases, and batch effect removal. Wang et al. (2018) processed data from GTEx and TCGA and addressed those issues to facilitate data comparison. They provided the normalized datasets on figshare (Wang et al., 2018). In this study, we downloaded 511 breast cancer samples and 212 normal samples for downstream analysis.

### Subgrouping Tumor Samples by Non-negative Matrix Factorization

Because of tumor heterogeneity, the molecular and cellular characteristics of a single tumor between different tumor patients (inter-tumor heterogeneity) may show significant differences. In this study, focusing on the tumor cells’ behavior of immune evasion, we clustered the 511 breast cancer samples with using non-negative matrix factorization (NMF; Gaujoux and Seoighe, 2010). For the completeness of the demonstration, in this section we briefly introduce NMF mathematical formulation. Formula (1) shows an approximation of a matrix *X* containing *n* features and *p* samples. Of note, all entries in *X* are non-negative.

(1) X≈W⁢H

where *W* is non-negative matrix containing *n* rows and *r* columns, *H* is a non-negative matrix containing *r* rows and *p* columns, and the factorization rank *r* is a positive integer. In this study, *r* is the number of subgroups, so that *W* denotes the weights of each feature contributing to each cluster and *H* represents the weights of each sample affiliating to each cluster.

Estimating the approximate solutions of *W* and *H* can be considered as an optimization problem in Formula (2).

(2) $$\min_{W,H\geq 0}\left|F-\mathrm{WH}\right|+\gamma \,R\,\left(W,H\right)$$

where the first component is used to measure the quality of the approximation, namely loss function. For avoiding overfitting, the second component uses a regularization function to ensure sparsity or smoothness of matrices *W* and *H*. γ is a parameter for balancing these two components.

The parameter *r*, namely factorization rank, represents the number of subgroups divided. For recognizing significantly different subgroups of breast cancer samples, a simple way is to try different values of *r* and then choose the best *r* according to quality measure of clustering results. For example, Frigyesi and Höglund (2008) chose *r* as the minimum factorization rank when the marginal reduction in residuals is still greater than the reduction observed for random data. The robustness of clustering can be evaluated the cophenetic correlation coefficient derived from a consensus matrix (Frigyesi and Höglund, 2008).

### Differential Expression Analysis

Comparing each cluster with normal samples, we can find which genes are differentially expressed. And these differential genes are likely to be very critical in tumor development and evolution. With using overdispersed Poisson model and empirical Bayes method, edgeR has shown superiority in terms of variability and robustness and has become a widely used Bioconductor software package for differential expression analysis (Robinson et al., 2010). The downstream analysis of high-throughput sequencing RNA-seq is confronted with a number of issues, such as systematic changes across experimental conditions, discreteness, and small replicate numbers. To address those problems, DESeq2 applied shrinkage estimation for dispersions and fold changes to improve stability and interpretability of estimates (Love et al., 2014). It can be found that those two methods use different statistical models and have different specificities. Genes identified as differentially expressed by both edgeR and DESeq2 have higher confidence. In order to eliminate false positives, we consider the candidate pathogenic genes as the consensus results of edgeR and DESeq2.

### Spearman’s Correlation Coefficient

Sharma et al. (2018) conducted a pan-cancer analysis on the relationships among cancer-associated genes’ expression variation, CNV, epigenetic changes, transcription factors, and microRNAs using a generalized linear model. They concluded that CNV is the most important factor contributing to the variation of gene expression. In this study, we propose to use Spearman’s correlation to examine the correlation between CNV and mRNA of candidate pathogenic genes. The Spearman’s correlation is a statistical method for measuring the strength of a monotonic relationship between two variables. Of note, its calculation and significance test are based on two assumptions: the data of two variables are interval or ratio level or ordinal and they are monotonically related. The value of Spearman’s correlation coefficient is in [−1, +1], and the closer to +1, the stronger the positive correlation; the closer to −1, the stronger the negative correlation.

### Regulatory Analysis Based on ATAC-seq

UCSC Xena provides the ATAC-seq peak signal for the 404 TCGA samples in Pan-cancer manner.^1^ In this study, all peaks locating with 20 kb from a gene’s TSS sites are considered as candidate regulatory region containing TF (transcription factor) or RP (repressor protein). The raw matrix data downloaded from UCSC Xena contains 562,710 identifiers and 404 samples and each unit is defined as “log2([count + 5]PM)-qn.” Note that 70 breast cancer samples out of 404 samples are available for immune gene regulatory analysis.

There are lots of peaks mapped to the target gene, so that a multi-objective optimization strategy is proposed to rank all peaks. Here, we use the following three evaluation criteria to examine every peak from different angles.

*Distance and score value-based criterion*: The closer to the target gene position, it indicates that the peak region has a greater possible regulatory relationship with the gene, and the higher the score of peak, it indicates that the RP or TF in this region is more active, and the region is likely to have a regulatory effect on the target gene.

*Spearman’s correlation-based criterion*: If there is a strong linear relationship between the variation of gene expression and the change of peak score value, then it can be considered that there is a certain regulatory relationship between target gene and TF or RP within the peak region. We apply the Spearman’s correlation coefficient to measuring the strength of regulatory relationship between target gene and TF or RP within the peak region. If the Spearman’s correlation coefficient is higher than 0.4 or lower than -0.4, there is a moderate relationship between the two at least. In this study, we have, first, designed a framework to analyze regulatory mechanism based on ATAC-seq data.

Regulatory analysis algorithm based on ATAC-seq:

Input: the mRNA of breast cancer samples and GTEx normal samples; the peak matrix and peak location information.

Output: differential expression immune-related genes’ TFs and RPs.

For each individual of breast cancer sample:

for each target gene:

Use the multi-objective evaluation criteria to select the candidate peaks, and then extract the position information of the chromosome where the peak locates;

*Use BEDTOOLS* (Quinlan and Hall, 2010) *to extract the DNA sequence corresponding to the candidate peaks on the reference genome hg38;*

Enter the DNA sequence in the PROMO website to get the possible TFs binding to the DNA sequence;

*Enter the sequence in the PROMO* (Messeguer et al., 2002) *to get the candidate TFs in the sequence. Then, search in the UNIPROT and GENECARD to determine the corresponding gene coding the candidate TFs;*

for each TF coding gene:

use the Student’s t-test to compare TF coding gene expression in single breast cancer sample with GTEx samples;

if high expression in breast cancer sample AND high TF peak score:

TF – target gene Positive regulation;

Endif

if low expression in breast cancer sample AND high RP peak score:

RP – target gene Negative regulation;

Endif

Endfor

Endfor

Endfor

Regarding the above algorithm, there are three points that need to be emphasized and further explained. First, it can be seen from the pseudo-code that our analysis of regulatory mechanism is at a single-sample level, which is conducive to further revealing the heterogeneity of tumors. Regarding whether the expression of the target gene is regulated up or down in a single sample analysis, our method is to carry out the Student’s t-test statistical analysis of the expression value of the gene with multiple normal samples. Second, regardless of whether it is positive regulation or negative regulation, only a high peak score can indicate that the region is in an open state and that it may be regulated by TF and RP. Last, the high expression of the gene encoding the regulator indicates that the regulator is likely to be involved in the regulation of the corresponding target gene. If the target gene is highly expressed, it means that there is a positive regulation between them; the low expression of the gene encoding the regulator indicates that the regulator is not involved in the regulation of the target gene. If the target gene is highly expressed at this time, it means that the original negative regulation effect of the regulator is inhibited.

## Results

### Clustering Breast Cancer Samples

**FIGURE 2:**
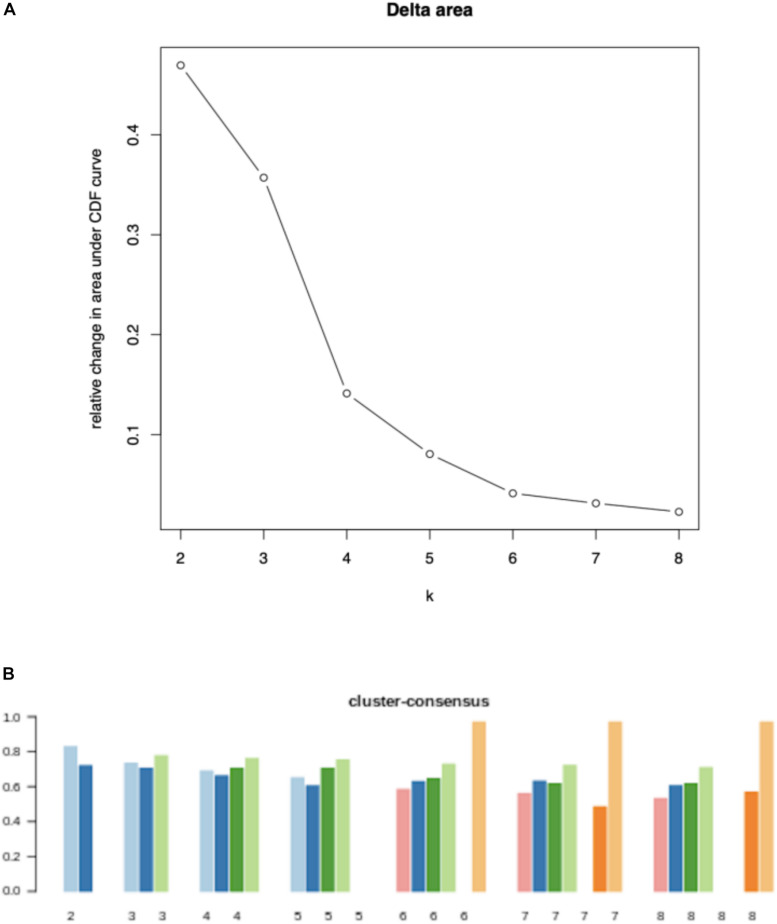
Quality measures for each value of factorization rank: Delta area **(A)** and cluster-consensus **(B)**.

To study the tumor heterogeneity, we used NMF to cluster 511 breast cancer samples according to the 2171 candidate immune-related genes. In Figure 2, the Delta area and cluster-consensus of different factorization rank values are depicted, which can help researchers to estimate the best rank value. We can find that when *k* = 4, clustering consistency and stability are relatively optimal. Therefore, we believe that there are 4 different immune subtypes in these 511 breast cancer samples, and the numbers of samples corresponding to each subtype are 199, 60, 169, and 83.

### Differentially Expressed Genes Among Subtypes

Different tumor subtypes may have different immune escape pathways. Understanding the differences in escape mechanisms between different subtypes is conducive to the implementation of precise immunotherapy. In order to understand the differences and commonalities of the dominant immune escape pathways among different subtypes, we compared the immune-related gene expression of each subtype with normal samples collected from GTEx.

Actually, to avoid the damage caused by NK cells, tumor cells have two possible strategies to suppress immune activity: one is to lower the expression of NK activator ligands, and the other is to increase the expression of NK inhibitor ligands. Table 1 lists all the differentially expressed NK ligand genes of 4 subtypes derived by the consensus of edgeR and DESeq2. The second column separately lists all these NK activator ligands in each cluster that have significantly lower expression than GTEx normal samples. The third column separately lists all these NK activator ligands in each cluster that have significantly higher expression than GTEx normal samples. The fourth column separately lists all these NK inhibitor ligands in each cluster that have significantly lower expression than GTEx normal samples. Of note, since no significantly low expressed genes were found in this case, we use the ’-’ symbol to indicate. The fifth column lists all these NK inhibitor ligands in each cluster that have significantly higher expression than GTEx normal samples.

**TABLE 1:** Differentially expressed genes of each subtypes.

Cluster	Low expressed NK activator ligands	High expressed NK activator ligands	Low expressed NK inhibitor ligands	High expressed NK inhibitor ligands
Cluster 1	*CFP, CLEC2B, CSF1, CSF3, HLA-E, IL15, TNFRSF10B, TNFRSF14, VIM*	*CADM1, CD48, CD70, CD80, CD86, CSF2, IL18, MICB, PCNA, RAET1E, RAET1L, SELL, SLAMF6, SLAMF7, ULBP1*,	–	*PVRL2, CEACAM5, CDH1*
Cluster 2	*CFP, CLEC2B, CSF1, CSF3, HLA-E, HSPG2, IL15, IL2, MICA, RAET1G, TNFRSF10B, TNFRSF14, ULBP3, VIM*,	*CADM1, CD48, CD80, CD86, CSF2, IL18, MICB, PCNA, RAET1L, SELL, SLAMF6, SLAMF7, ULBP1*	–	*PVRL2, CEACAM5, CDH1*
Cluster 3	*CLEC2B, HLA-E, IL15, MICA, PDCD1LG2, TNFRSF14, VIM*	*CADM1, CEACAM1, PCNA, RAET1E, SELL, ULBP1*	–	*CEACAM1, PVRL2*
Cluster 4	*CFP, CLEC2B, CSF1, CSF3, HLA-E, IL15, MICA, TNFRSF10B, TNFRSF14, ULBP3, VIM*	*CADM1, CD48, CD80, CD86, CSF2, IL18, MICB, PCNA, RAET1L, SELL, SLAMF6, SLAMF7, ULBP1*	–	*PVRL2, CEACAM5, CDH1*

From the results of NK activator ligands expression shown in Table 1, it can be found that the NK activator ligands among clusters 1, 2, and 4 are quite similar. Although they are similar, there are also some differentially expressed NK activator ligands. These genes may be the key to different subtypes of immune escape pathways. A possible hypothesis of tumor development is that NK activator ligands are regulated to lowly express, which makes it difficult for tumor cells to be destroyed. *CLEC2B*, *HLA-E*, *IL15*, and *VIM* all appear in the 4 subtypes at the same time, which may mean that they play a more general role in different subtypes of breast cancer. However, it is very interesting that the results of NK inhibitor ligands between different clusters are very similar. Note that *CDH1* and *PVRL2* appear in all clusters at the same time, which probably means that they play a very important role in the immune evasion of breast cancer. Therefore, it is necessary to further study how tumors regulate *CDH1* and *PVRL2* to escape the destruction of NK cells.

### The Correlation of CNV and mRNA on *CDH1* and *PVRL2*

In this section, the Spearman’s correlation coefficient is used to measure the correlation strength between the ligand gene itself CNV and mRNA. If the correlation strength between them is strong, it can explain that the change in mRNA is caused by itself CNV, otherwise it means that there may be other regulatory factors in the change of mRNA.

For *CDH1*, its CNV and mRNA have moderate Spearman’s correlation coefficient (correlation value 0.54, *P*-value adjusted: 3.12e-05), which means that the variation of *CDH1*’s expression can be moderately explained by its CNV. However, for *PVRL2*, the correlation is very weak, so that there must be other factors regulating the gene expression of *PVRL2*.

### The Regulatory Mechanism Analysis Based on ATAC-seq

**FIGURE 3:**
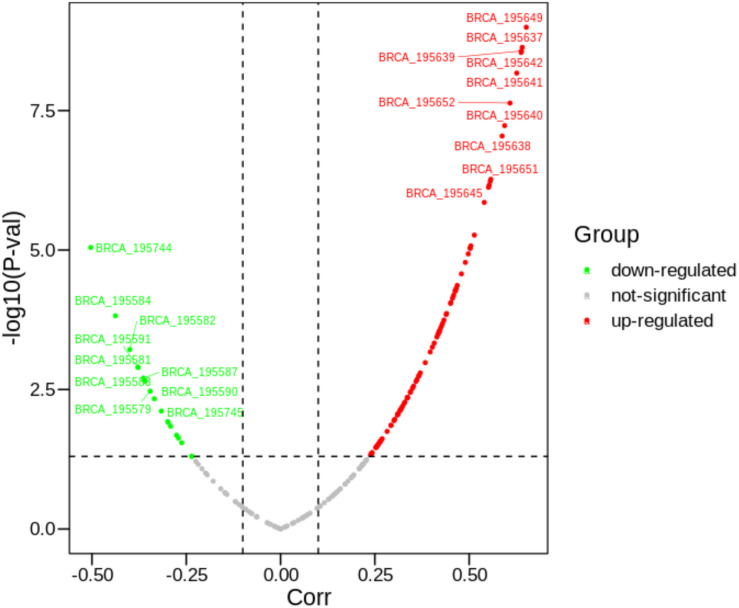
The correlations between 190 peaks with *PVRL2*. The text attached to the curve indicates the ID of the peak.

As mentioned above, we further explored what factors are regulating the expression changes of *PVRL2*. As shown in Figure 3, it can be found that there are about 190 peaks around *PVRL2*. Some peaks have significantly negative correlation with *PVRL2*, while some have positive correlation. With using our multi-objective criterion for peak selection, three candidate peaks (the most positive correlation peak, the most negative peak, and the closest gene with the highest score peak) are reserved for downstream analysis. Then, according to the regulatory analysis algorithm, 10 TFs (*AR, ESR1, FOXP3, GTF2I, NR3C1, PAX5, SP1, TFAP2A, TP53*, and *YY1*) have been considered to potentially regulate the gene expression of *PVRL2*.

To verify the real existence of that regulatory relationship, we matched 5 commonly used databases, namely JASPAR (Portales-Casamar et al., 2010), ENCODE (Encode Project Consortium, 2011), ChEA (Achmann et al., 2010), MotifMap (Daily et al., 2011), and TRANSFAC (Matys et al., 2003). The validation results are listed in Table 2; the “√” indicates that the corresponding TF has a regulatory relationship with *PVRL2* and “–” indicates that the database does not include the corresponding regulatory relationship between TF and *PVRL2*.

**TABLE 2:** The validation correlation between TFs and *PVRL2*.

TF	JASPAR	ENCODE	CHEA	MotifMap	TRANSFAC
AR	–	–	–	–	–
ESR1	√	√	–	–	–
FOXP3	–	–	–	–	–
GTF2I	–	–	–	–	–
NR3C1	–	√	–	–	–
PAX5	–	√	–	–	–
SP1	–	√	–	–	–
TFAP2A	√	–	–	–	√
TP53	–	–	–	–	–
YY1	–	√	–	√	–

From Table 2, we can find that the six TFs were confirmed by the databases to have corresponding regulatory relationships with *PVRL2*. More importantly, all these TFs show high expression in most samples (>50%). Thirty-seven breast cancer samples, namely more than half of the breast samples, show amplification with CNV, which means that most of breast cancer samples’ TF coding gene are amplified by CNV, which leads to the high expression of TF gene, thereby positively regulating the *PVRL2*, so that the expression of the *PVRL2* also increases, thereby inhibiting the activity of NK cells and achieving immune escape. But for those samples where the TF gene does not show high expression, they may have more complex regulatory mechanisms, or there are still some TFs that have not been confirmed.

For the *CDH1*, the same regulatory analysis pipeline is also applied. TFs such as *AR, GTF2I, IRF2, NF1, NFATC2, XBP1*, and *YY1* show significant correlation with *CDH1*. Then, these relationship between TFs and *CDH1* are also searched in JASPAR, ENCODE, ChEA, MotifMap, and TRANSFAC databases, and the results are listed in Table 3.

**TABLE 3:** The validation correlation between TFs and *CDH1*.

TF	ASPAR	ENCODE	CHEA	MotifMap	TRANSFAC
AR	–	–	√	–	–
GTF2I	–	–	–	–	–
IRF2	–	–	–	–	–
NF1	–	–	–	–	–
NFATC2	–	–	–	–	–
XBP1	–	–	–	–	–
YY1	–	√	–	–	–

The results in Table 3 show that only 2 TFs have reported to be correlated with *CDH1.* At the same time, approximately one-third of the samples show amplification of TFs. This means that in these one-third of the samples, it may be due to the high expression of these TFs caused by itself CNV amplification, which positively regulates the high expression of these target genes. For the remaining two-thirds of the samples, the high expression of *CDH1* may be due to a more complicated mechanism, or, as mentioned above, *CDH1* is more likely to be caused by its own CNV amplification. Consequently, combining the analysis results of the relationship between CNV and mRNA may better explain the variations of the *CDH1* expression.

### Survival Analysis of *CDH1* and *PVRL2*

**FIGURE 4:**
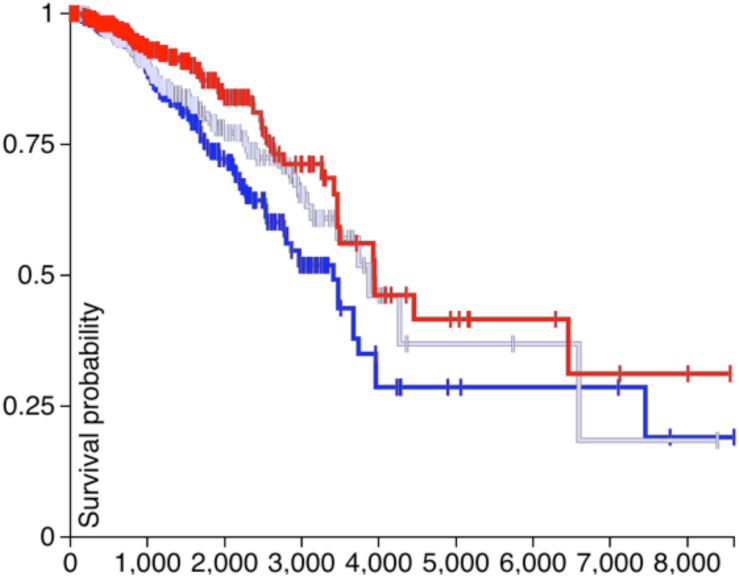
The survival results of *PVRL2*.

**FIGURE 5:**
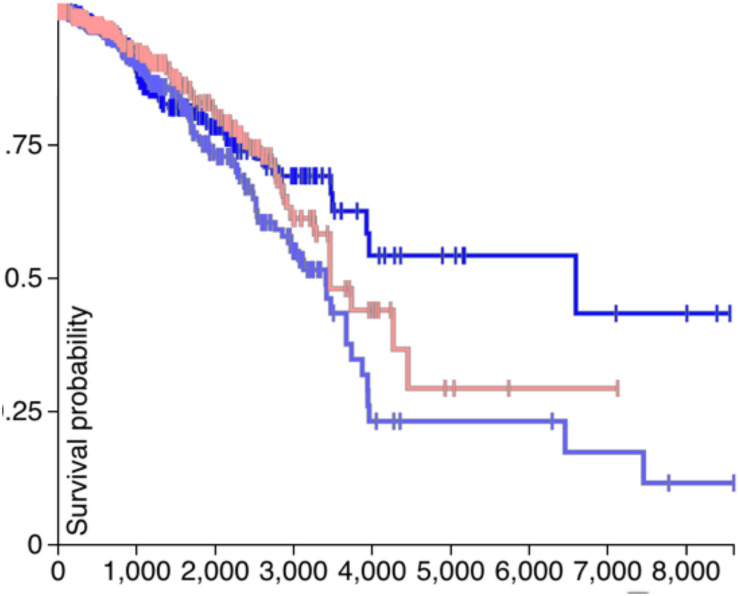
The survival results of *CDH1*.

If the *CDH1* and *PVRL2* are essential for tumor cells avoiding immune evasion, their expression should affect the survival of patients. Therefore, in Figures 4, 5, the survival analysis results of are *CDH1* and *PVRL2* depicted.

In Figure 4, the blue line denotes low expression of *PVRL2*, the gray line means moderate expression of *PVRL2*, and the red line represents high expression of *PVRL2*. The P-value is 0.009, so that *PVRL2* significantly influences the breast cancer patient’s survival quality.

For *CDH1*, in Figure 5, the dark blue line denotes low expression, the light blue line means moderate expression, and the red line represents high expression. The P-value is 0.05, and although the results of statistical testing are not very significant, there may be a certain relationship between *CDH1* and breast cancer.

## Discussion and Conclusion

A large number of studies have confirmed that the occurrence and development of complex diseases such as tumors usually involve the interaction of multiple factors such as the environment and genetic mutations. However, it is difficult for a single level of omics data to systematically and completely reveal how multiple factors interact. At the same time, single-source data sets are usually limited by factors such as the sample population, sample size, and data type, resulting in insufficient statistical power and difficulty in repeating association studies. Therefore, the research in this study provides an analysis framework that integrates multi-source data, which can effectively enhance the biological meaning of the research process and research results. More importantly, the single-sample regulation analysis method of this study can explore the heterogeneity of tumors in more depth, which is of great significance to the practice of precision medicine. Understanding tumor heterogeneity (inter-tumor heterogeneity or intra-tumor heterogeneity) is an important foundation for precision medicine. This is because different subtypes may use completely different immune escape pathways. If the same treatment is used, it may not only have no effect but also cause side effects. In this study, we proposed a multi-source fusion framework for understanding the immune evasion mechanism of breast cancer. Our method has three main characteristics:

1. Collect immune-related genes and combine TCGA case samples and GTEx normal samples to identify specific immune genes associated with different subgroups of breast cancer. Using this strategy, there are lots of potential NK activator ligands in low expression and NK inhibitor ligands in high expression, which may play a key role in immune evasion are identified.
2. Design a multi-objective criterion for evaluating the importance of peaks nearby target gene and propose a regulatory analysis algorithm to locate TFs or RPs regulating the expression of target immune-related genes based on ATAC-seq data.
3. We have explained the target gene expression variations on single sample level, which shows that the framework designed in this study can serve precision medicine. We use the statistical method (Student’s *t*-test) to identify whether the expression of the target gene on a single sample is higher than its expression in normal samples (GTEx), and then analyze the specific reasons (CNV or regulators) for the target gene’s expression variations with a single sample manner.

In this study, we found that both *CDH1* and *PVRL2* show high expression in all subgroups and they are NK cell inhibitor ligands, so that their high expression may play an important role in immune evasion. Previous studies concluded that CDH1 mutation contributes to the risks of breast cancer (Shabnaz et al., 2016; Xicola et al., 2019). For *CDH1*, we found that its expression variation is mainly caused by its own CNV. In addition, *CDH1* may also be positively regulated by some TFs. Whelan et al. concluded that *PVRL2* is expressed in human cancers and the PVRIG–PVRL2 pathways are non-redundant inhibitory signaling pathways (Whelan et al., 2019). For *PVRL2*, we found that its CNV and mRNA have very weak correlation. More than half of the samples showed high expression of TFs, which means that the high expression of *PVRL2* may be caused by high expression of TFs. The relationship of *PVRL2* and TFs is validated in relevant databases.

Although this study found some genes that are differentially expressed in diseased samples, only two common differential expression genes, namely *CDH1* and *PVRL2*, have in-depth discussions on their regulatory mechanism. This study aims to clarify the practicality of the fusion analysis framework. In future work, we will further study the regulatory mechanisms of other important differential genes in each single breast cancer sample and will also study the role of these immune-related genes in the occurrence and development of tumors with a pan-cancer manner.

## Data Availability Statement

Publicly available datasets were analyzed in this study. This data can be found here: https://portal.gdc.cancer.gov/.

## Author Contributions

XC, HC, and LC conveyed the conceptual idea and carried out the design of the study. YL and QQ implemented the experiments and analyzed the results. YL, QQ, and BN performed the statistical analysis and helped to draft the manuscript. All authors read and approved the final manuscript.

## Conflict of Interest

The authors declare that the research was conducted in the absence of any commercial or financial relationships that could be construed as a potential conflict of interest.

## Footnotes

1 https://atacseq.xenahubs.net
